# Polyethyleneimine‐Based Cryogels Enabling the Selective and Reversible Adsorption of Chlorine

**DOI:** 10.1002/advs.202414274

**Published:** 2024-12-30

**Authors:** Alejandro Lorente, Johanna S. Sturm, Merlin Kleoff, Fabio Lorenz, Patrick Voßnacker, Olaf Wagner, Rainer Haag, Sebastian Riedel

**Affiliations:** ^1^ Freie Universität Berlin Institut für Chemie und Biochemie – Organische Chemie Takustr. 3 14195 Berlin Germany; ^2^ Freie Universität Berlin Institut für Chemie und Biochemie – Anorganische Chemie Fabeckstr. 34/36 14195 Berlin Germany

**Keywords:** adsorption, chlorine, cryogels, polychlorides, polymers

## Abstract

Herein hyperbranched polyethyleneimine (hPEI) cryogels are reported for the selective and reversible adsorption of elemental chlorine. The cryogels are prepared in an aqueous solution by crosslinking with glutaraldehyde at subzero temperatures. The final macroporous composites bearing ammonium chloride groups are obtained after freeze‐drying. The cryogels CG1[Cl]–CG3[Cl] adsorb chlorine with capacities of 0.22–0.26 g Cl_2_/g cryogel as an average over three adsorption‐desorption cycles. The adsorption process is based on the reversible and selective halogen bonding of chlorides (Cl^−^) with chlorine (Cl_2_) forming the corresponding trichloride ([Cl_3_]^−^) species, indicated by Raman spectroscopy. The reversibility of chlorine adsorption is shown by applying heat and vacuum to the loaded cryogel CG1[Cl_3_] releasing 63% of the adsorbed chlorine within 3 h and 72% within 16 h. The unique ability to selectively adsorb chlorine in the presence of other gases is successfully employed for the selective adsorption of chlorine from a gas mixture, potentially enabling the recycling of chlorine from tail gas streams.

## Graphical abstract

Quaternized polyethyleneimine‐based cryogels **CG[Cl]** are used for the reversible adsorption of chlorine. The chlorine is adsorbed by chemisorption forming the corresponding trichlorides **CG[Cl_3_]** by halogen bonding. Using these cryogels, chlorine is selectively adsorbed in the presence of oxygen and nitrogen potentially enabling gas separation processes in the chemical industry.

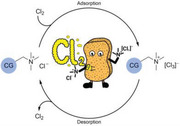

## 1 Introduction

Chlorine (Cl_2_) is one of the most important base chemicals, with a production scale of 100 million tons in 2023.^[^ ^1^ ^]^ In fact, it is involved in the production of more than 50% of all industrial chemicals and polymers, 30% of all agrochemicals, and 20% of all pharmaceuticals.^[^ ^2^ ^]^ Despite the enormous importance of chlorine, its utilization is associated with several drawbacks due to its toxicity and high reactivity.^[^ ^3^, ^4^ ^]^ Therefore, the handling and storage of chlorine gas is inherently dangerous as it has been witnessed by countless severe accidents.^[^ ^5^ ^]^ To overcome this problem, Riedel and coworkers recently developed a trichloride‐based technology for the safe storage and transport of chlorine. This technology utilizes the abundant triethylmethylammonium chloride[NEt_3_Me]Cl as a storage system which forms the corresponding trichloride [NEt_3_Me][Cl_3_] when exposed to chlorine gas. In this form it is an ionic liquid at room temperature^[^ ^6^ ^]^ and can be safely stored or used directly as a versatile chlorination agent.^[^ ^4^ ^]^

This ionic liquid system offers several advantages for the safe storage and transport of chlorine as well as for the production of base chemicals such as phosgene (COCl_2_).^[^ ^7^ ^]^ However, there are several industrial applications that profit from solid adsorbers for separation and purification steps.^[^ ^8^, ^9^, ^10^, ^11^ ^]^ Particularly, the selective adsorption of chlorine by solid adsorbers could enable the separation of chlorine from chlorine‐containing gas mixtures. These gas mixtures are formed in vast amounts for example in the chloralkali‐electrolysis as tail gas streams consisting primarily of chlorine and air.^[^ ^12^, ^13^, ^14^ ^]^ In this respect, a polymer system adopting the trichloride technology would be a complementary approach to safely store and process chlorine gas.

In recent decades, cryogels have evolved as versatile materials ranging from biomedical applications,^[^ ^15^, ^16^ ^]^ to the adsorption of gases such as CO_2_,^[^ ^17^ ^]^ heavy metals, anions, oil or dyes from water.^[^ ^18^, ^19^, ^20^, ^21^ ^]^ Thus far, the investigation of cryogels has been focused primarily on small scale applications due to the limited scalability when prepared in batch. However, recently it was demonstrated that the application of flow chemistry enables a scalable synthesis of cryogels with defined properties.^[^ ^22^, ^23^, ^24^, ^25^ ^]^ With this key technology, new applications of cryogels can be explored.^[^ ^22^, ^23^, ^26^ ^]^

## 2 Results and Discussion

At the outset of this work, we envisioned to synthesize a family of cryogels that can selectively adsorb chlorine from chlorine‐containing gas mixtures and allow reversible chlorine adsorption. Due to the high reactivity of chlorine, we anticipated that hyperbranched polyethyleneimine (hPEI) having no additional functional groups besides amines, would serve as a suitable polymer backbone.^[^ ^27^, ^28^, ^29^ ^]^

**Scheme 1:**
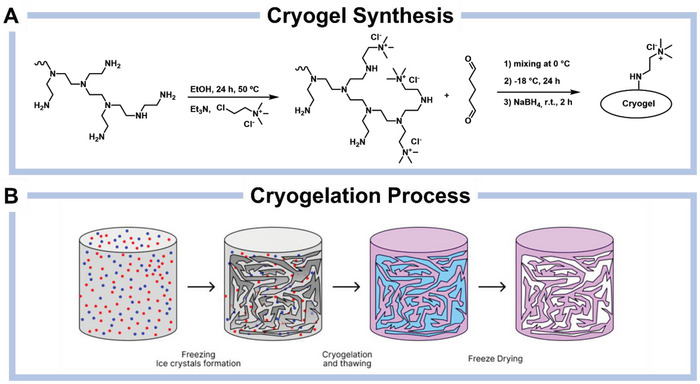
A) The general scheme of the synthesis of **PEICCC** precursor and crosslinking reaction with glutaraldehyde and B) the cryogelation process.

This polymer has a 2:1 ratio of C to N atoms rendering this material a backbone with a high number of sites that are easy to functionalize. To introduce the quaternary ammonium chloride functionality, hPEI was alkylated with chlorocholine chloride (CCC). The composition of the resulting polymer was studied by IR (Figure S5, Supporting Information), ^1^H‐NMR (Figure S2, Supporting Information) and ^13^C{^1^H}‐NMR (**Scheme** **1**).

**Figure 1:**
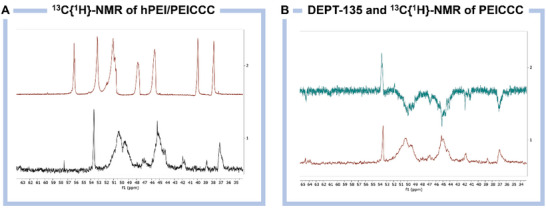
A) Inversed‐gate ^13^C{^1^H}‐NMR spectra of **hPEI** (red) and **PEICCC** (black). B) DEPT‐135 and ^13^C{^1^H}‐NMR of **PEICC**.

The most significant results were obtained by ^13^C{^1^H}‐NMR (**Figure** **1**). As described elsewhere, the composition of commercial hPEI can be characterized by quantitative inversed‐gate ^13^C{^1^H}‐spectra.^[^ ^30^, ^31^, ^32^ ^]^ By observing the different substitution patterns in hPEI's general structure, up to 8 different types of carbons can be seen, corresponding to different α‐β nitrogen configurations (Figure S3, Supporting Information), this allows to estimate the composition of our unmodified hPEI with a primary/secondary/tertiary amine ratio of 34.0/33.2/32.8, respectively. After functionalizing hPEI with CCC, the same strategy was used to estimate the degree of substitution in **PEICCC**. However, the ^13^C{^1^H}‐NMR peaks do not clearly indicate the different carbons. Nevertheless, using DEPT‐135, the (CH_3_)_3_ group of the quaternary ammonium is identified at 53.6 ppm (Figure S4, Supporting Information). The degree of substitution (DS) is estimated to be 15% for the whole polymer backbone (see methods in Supporting Information). Notably, a partial substitution of the 1° and 2° amines was intended for later use in the crosslinking reaction with glutaraldehyde (GA).

Several types of crosslinkers can be used for a cryogel preparation.^[^ ^19^, ^33^ ^]^ GA is the most suitable for our purposes, as it condenses with the free 1° and 2° amino groups of **PEICCC** and, after reduction with NaBH_4_, forms 2° or 3° amines that are less likely to undergo side reactions with Cl_2_. As described by Sahiner et al., crosslinking hPEI with glutaraldehyde typically requires pH 4.6 to prevent premature reactions.^[^ ^20^ ^]^ In our case, we reduced the reaction rate by lowering the temperature of **PEICCC** and GA solutions to 0–1 °C (for feed compositions see Table S1, Supporting Information). After 24 h at −20 °C, the formed cryogels were thawed, washed with 0.2% aqueous solution of NaBH_4_, refrozen, and finally obtained after freeze‐drying.

To tailor the interconnected macroporous structure of the cryogels, we varied the initial PEICCC concentration: 2 wt.% for **CG1[Cl]**, 5 wt.% for **CG2[Cl]**, and 10 wt.% for **CG3[Cl],** respectively. By adjusting the PEICCC concentration, while keeping all other parameters constant, we synthesized a series of cryogels with increasingly dense macroporous structures.

**Figure 2:**
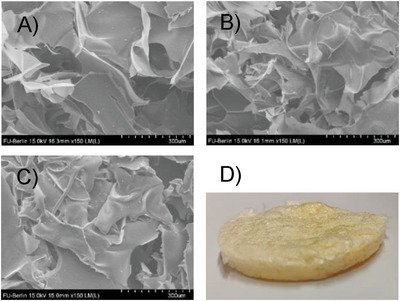
SEM images at 150 magnifications for **CG1[Cl]** A), **CG2[Cl]** B), **CG3[Cl]** C), and the physical appearance of the cryogels after the final drying process D). Photographs taken by the authors.

The morphology of cryogels was characterized by Scanning Electron Microscopy (SEM) and mercury intrusion porosimetry. A rather irregular pattern of interconnected macropores can be observed for all cryogels (**Figures** **2** and S7, Supporting Information). This is most likely due to the irregular formation of ice crystals that grow during the freezing processes. However, it can also be observed that the porous structure is more densely packed when a more concentrated solution of PEICCC is used. These observations are also supported by the mercury intrusion porosimetry measurements. As can be seen in Table S2 (Supporting Information), the median pore volume ranges from 102 µm for **CG1[Cl]** to 93 µm for **CG3[Cl]** which is in agreement with the use of more diluted starting solutions of PEICCC. This is similarly observed in the case of the modal pore diameter which is in the range from 105 µm for **CG1[Cl]** and 97 µm for **CG3[Cl]**.

The thermal stability of cryogels **CG1[Cl]‐CG3[Cl]** was studied by thermogravimetric analysis (Figure S6, Supporting Information). The curves show three transitions. The first significant mass loss of 11–14% occurs between 30 and 100 °C, likely due to a water film on the hygroscopic surface. A second plateau‐like section appears between 100 and 250 °C, with a mass loss of 8% for **CG1[Cl]** to 11% for **CG3[Cl]**. Above 250 °C, a rapid mass loss occurs due to the decomposition of the crosslinked polymer structure.

**Figure 3:**
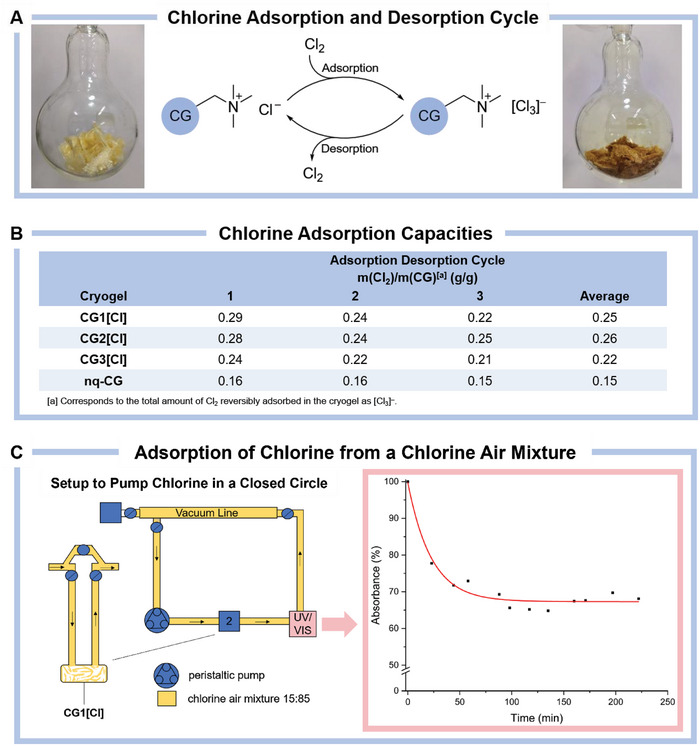
A) Chlorine adsorption and desorption cycle. The cryogel chloride **CG[Cl]** (left) adsorbs chlorine forming the corresponding cryogel trichloride **CG[Cl_3_]** while turning dark yellow (right). B) Chlorine adsorption capacities of the cryogels **CG1[Cl]**–**CG3[Cl]** and the non‐quaternized PEI‐cryogel **nq‐CG** for three chlorine adsorption and desorption cycles and the averaged value of three cycles. C) Setup for the selective adsorption of chlorine by the cryogel **CG1[Cl]** from a gas mixture of chlorine and air (15:85; left). The gas mixture was pumped in circuit using a peristaltic pump and the chlorine content was measured by UV/VIS spectroscopy over time showing a rapid, initial decrease of the chlorine content while reaching slowly a saturation of the cryogel **CG1[Cl]**. The absorbance of 100% corresponds to the initial chlorine content of 15% (right). Photographs taken by the authors.

With the cryogels **CG1[Cl]**–**CG3[Cl]** in hand, we investigated their ability to adsorb elemental chlorine. We noted that upon first contact with chlorine the cryogels react partially with chlorine probably due to the chlorination of N─H or C─H bonds in the structure. Therefore, prior to their use, the cryogels were conditioned with chlorine gas over 16 h followed by chlorine removal under reduced pressure (see the Section S3.1, Supporting Information). Next, the cryogels **CG1[Cl]**–**CG3[Cl]** were exposed to an atmosphere of chlorine, and the chlorine adsorption capacities were determined gravimetrically (see **Figure** **3**A and the Section S3.2, Supporting Information). Remarkably, all three cryogels **CG1[Cl]**–**CG3[Cl]** show similar chlorine adsorption capacities of 0.24–0.29 g Cl_2_/g cryogel. Subsequently, the chlorine was removed under reduced pressure completing one adsorption desorption cycle. This process was repeated for two more cycles. When averaged over three cycles, the cryogels **CG1[Cl]**–**CG3[Cl]** show chlorine adsorption capacities in the range of 0.22–0.26 g Cl_2_/g cryogel. Based on these results, all three cryogels **CG1[Cl]**–**CG3[Cl]** could be applied as chlorine adsorption matrices, thus, further studies were focused on cryogel **CG1[Cl]** as a representative example of these materials.

Interestingly, we found that the non‐quaternized cryogel **nq‐CG** is also able to adsorb chlorine to a lower extend of 0.15 g Cl_2_/g. This is probably a result of the significant reaction of the cryogel **nq‐CG** with chorine releasing HCl that can pair with free amines present in the cryogel forming the corresponding ammonium chlorides [NR_3_H][Cl]. Thus, supposedly, the non‐quaternized cryogel **nq‐CG** undergoes quaternization when coming in contact with chlorine and therefore shows a moderate chlorine adsorption capability, as well.

To investigate the long‐term stability of the cryogels, elemental analyses of **CG1[Cl]** were performed before and after three adsorption‐desorption cycles. The composition of **CG1[Cl]** changes only moderately despite exposition toward chlorine repeatedly and for prolonged times (see the Section S3.2.2, Supporting Information).

**Figure 4:**
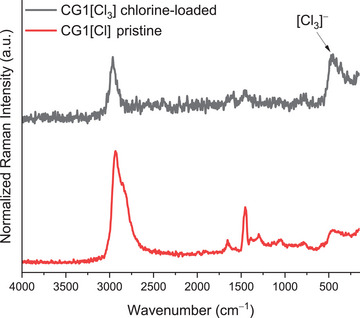
Raman spectrum of pristine **CG1[Cl]** (red) and the chlorine‐containing cryogel **CG1[Cl_3_]** (black).

Next, we investigated the cryogel **CG1[Cl]** before (**Figure** **4**, red) and after exposing it to chlorine by Raman spectroscopy (Figure 4, black). Both spectra show bands with similar wavenumbers in the range of asymmetric C─H stretching between 2933 and 2964 cm^−1^.^[^ ^34^ ^]^ Remarkably, in the cryogel that has adsorbed chlorine, another band of high intensity is present centered at 453 cm^−1^, which is typical for the trichloride monoanion [Cl_3_]^−^ as it has been previously described (see also the Section S3.2.3, Supporting Information).^[^ ^6^ ^]^

These observations indicate that the cryogels **CG1[Cl]**–**CG3[Cl]** form the corresponding cryogel trichlorides **CG1[Cl_3_]**–**CG3[Cl_3_]** upon contact with chlorine.

The trichloride anion [Cl_3_]^−^ can be imagined consisting of a chloride anion Cl^−^ and a Cl_2_ molecule. As the Cl_2_ molecule has an area of low electron density (a so‐called σ‐hole) along its Cl─Cl bond axis, it can act as a Lewis acid. The Lewis basic Cl^−^ can donate electron density into the σ‐hole of the dichlorine molecule forming the trichloride [Cl_3_]^−^.^[^ ^35^ ^]^

As this so‐called halogen bonding is a relatively weak interaction, the reaction of the cryogels **CG[Cl]** with chlorine gas forming the corresponding cryogel trichloride **CG[Cl_3_]** can be described as an equilibrium reaction (Equation 1).

(1) $$\mathrm{CG}\left[\mathrm{Cl}\right]+Cl_{2}⇌\mathrm{CG}\left[Cl_{3}\right]$$

This means in turn, that the cryogel trichlorides **CG[Cl_3_]** exist in an equilibrium with its chloride form **CG[Cl]** and gaseous chlorine, causing a low chlorine vapor pressure of the cryogel trichlorides **CG[Cl_3_]**. Therefore, by applying moderate heat and vacuum, the adsorbed chlorine can be released in a controlled way from the cryogel trichlorides **CG[Cl_3_]** while regenerating **CG1[Cl]** which is in agreement with our previous studies to the ionic liquid [NEt_3_Me][Cl_3_].^[^ ^6^ ^]^ At 60 °C and under a reduced pressure of 10^−3^ mbar, **CG1[Cl_3_]** released 63% of the adsorbed chlorine within 3 h, and 72% within 16 h, as determined gravimetrically (see the Section S3.2.1, Supporting Information). These results further support the hypothesis that the cryogels react with chlorine reversibly by chemisorption involving trichloride species.

As this adsorption process by halogen bonding is specific for halogens and pseudohalogens,^[^ ^35^, ^36^ ^]^ we anticipated that cryogel **CG1[Cl]** could be used for the selective adsorption of chlorine in the presence of other gases and therefore become useful for gas separation processes. Indeed, when exposing **CG1[Cl]** to nitrogen or oxygen no adsorption of these gases could be detected (see the Section S3.3, Supporting Information).

Based on these results, we tested the capability of the cryogel **CG[Cl]** to selectively adsorb chlorine from chlorine‐containing gas mixtures. Therefore, we constructed a setup to pump a mixture of chlorine and air (15:85) in a closed circuit (see the Section S3.4, Supporting Information). In this setup, the chlorine‐containing gas mixture was pumped through the cryogel **CG1[Cl]** and the chlorine concentration was consistently measured by UV/VIS spectrometry. In the first 30 min, a significant decrease of the chlorine concentration was observed, while the cryogel was saturated with chlorine reaching a final value of 70% of the initially pumped chlorine content after 4 h. This experiment highlights the ability of the cryogel **CG1[Cl]** to selectively adsorb chlorine from chlorine‐containing gas mixtures and could be employed in industrial gas separation processes.

## 3 Conclusion

In conclusion, we presented a strategy to translate the established trichloride chemistry of quaternary ammonium salts to polymeric adsorber materials. This was achieved by the synthesis of a family of quaternary ammonium cryogels **CG1[Cl]–CG3[Cl]** derived from hyperbranched polyethyleneimine (hPEI). The cryogels show reversible and consistent chlorine adsorption capacities of 0.22–0.26 g Cl_2_/g cryogel as an average over three adsorption cycles. Raman spectroscopy indicates the formation of trichloride species [Cl_3_]^−^ in the cryogel upon contact with chlorine. By applying heat and vacuum to the chlorine‐loaded cryogel **CG1[Cl_3_]**, 63% of the adsorbed chlorine was released within 3 h and 72% was adsorbed within 16 h. Finally, the cryogel **CG1[Cl]** was successfully used for the selective adsorption of chlorine from a chlorine air mixture. This could enable the separation of chlorine from chlorine‐containing gas mixtures allowing both the purification of air and the recycling of chlorine lost by tail gas streams and therefore contribute to an improved and more sustainable chemical industry. This technology is currently being further investigated in our laboratories.

## Conflict of Interest

The authors declare no conflict of interest.

## Supporting information



Supporting Information

## Data Availability

The data that support the findings of this study are available in the supplementary material of this article.
