# Supplementary material for: Polyethyleneimine‐Based Cryogels Enabling the Selective and Reversible Adsorption of Chlorine
Source: Adv Sci (Weinh). 2024 Dec 30;12(8):2414274. doi: 10.1002/advs.202414274 (PMC11848583; doi:10.1002/advs.202414274)
Supplement: Supplementary file 1 — Supporting Information [file ADVS-12-2414274-s001.docx]

Supporting Information:

Polyethyleneimine-based Cryogels Enabling the Selective and Reversible Adsorption of Chlorine

Alejandro Lorente,^[a]^ Johanna S. Sturm,^[b]^ Merlin Kleoff,^[b]^ Fabio Lorenz,^[b]^ Patrick Voßnacker,^[b]^ Olaf Wagner, ^[a]^ Rainer Haag,*^[a]^ and Sebastian Riedel*^[b]^

[a] Dr. A. Lorente Sánchez, Dr. O. Wagner, Prof. Dr. R. Haag

Freie Universität Berlin

Institut für Chemie und Biochemie - Organische Chemie Takustr. 3, 14195 Berlin

E-mail: haag@zedat.fu-berlin.de

[b] J. S. Sturm, Dr. M. Kleoff, F. Lorenz, Dr. P. Voßnacker, Prof. Dr. S. Riedel

Freie Universität Berlin

Institut für Chemie und Biochemie - Anorganische Chemie Fabeckstr. 34/36, 14195 Berlin

E-mail: s.riedel@fu-berlin.de

Supporting information for this article is given via a link at the end of the document.

**Table of Content**

[1. Materials and Methods 3](#_Toc178330693)

[1.1 Materials 3](#_Toc178330694)

[1.2 Methods 3](#_Toc178330695)

[2. Cryogel Synthesis and Characterization 5](#_Toc178330696)

[2.1 Synthesis of PEI-CCC 5](#_Toc178330697)

[2.2 Synthesis of Cryogels 1-3 (CG1[Cl]- CG3[Cl]) 8](#_Toc178330698)

[2.3 Characterization of Cryogels 9](#_Toc178330699)

[2.3.1 Infrared Spectroscopy 9](#_Toc178330700)

[2.3.2 Thermogravimetric analysis 10](#_Toc178330701)

[2.3.3 Scanning Electron Microscopy 11](#_Toc178330702)

[3. Chlorine Adsorption Experiments 12](#_Toc178330703)

[3.1 Conditioning of the Cryogels 12](#_Toc178330704)

[3.2 Chlorine Adsorption and Desorption Cycles 12](#_Toc178330705)

[3.2.1 Chlorine Release Process 13](#_Toc178330706)

[3.2.2 Elemental Analysis 14](#_Toc178330707)

[3.2.3 Raman Measurements 14](#_Toc178330708)

[3.3 Gas Adsorption Experiments with Nitrogen and Oxygen 15](#_Toc178330709)

[3.4 Separation Experiments 15](#_Toc178330710)

# Materials and Methods

## Materials

Polyethyleneimine, branched (99%, $\bar{M}$_w_ = 10000 gmol^–1^) was purchased from Polysciences Inc. Glutaraldehyde (50 wt%) was purchased from Thermo Scientific. Sodium Borohydrate (NaBH_4_ powder, 98+%) was purchased from Acros Organics. Chlorocholine chloride (98%) was purchased from TCI Chemicals. Elemental chlorine was purchased from Linde (Cl_2_, 99.8%). Elemental oxygen (O_2_, 99.999%) and nitrogen (N_2_, 99.999%) were purchased from AirLiquide (O_2_, 100%). Compressed air was dried before use. All other materials and solvents used were of technical grade.

## Methods

**IR** spectra were recorded using a Spectrum Two FT-IR spectrometer by PerkinElmer. The measuring range was set between 4000 cm^−1^ to 400 cm^−1^.

**SEM** measurements were conducted using a Hitachi SU8030 field emission scanning electron microscope (FE-SEM) from Japan. The SEM was operated at an accelerating voltage of 15 kV and a current of 10 μA. Prior to SEM imaging, the samples were lyophilized and coated with a 5 nm layer of gold using an Emscope SC 500 sputter coater from Quorum Technologies, UK. The sputter coating process took place for 30 seconds under an argon atmosphere, with a current of 30 mA and a pressure of 10^−1^ Torr (1.3 mbar).

**TGA** was performed on a STA 449 F3 Jupiter instrument from NETZSCH, in ceramic crucible, where around 12 mg of the cryogels were used. The samples were analyzed using a 10 K/min ramp rate from 30 °C to 800 °C. The data was plotted using Origin 9.0 software.

For the **tangencial flow filtration (TFF)** used for purification of the polymers a QF15QCon pump was used, connected to a Sartocon Slice filterholder that was equipped with a 5 kDa hydrostar 0.1 m² cassette. The crude of the reaction was dissolved in 800 mL of deionized water and the process run for ca. 3 hours.

For **mercury intrusion porosimetry (MIP)** measurements an AutoPore V instrument of the company Micromeritics Instrument Corporation was used. The measurements in the low-pressure range were measured from 0.0036 MPa until 0.3 MPa and the high-pressure measurements until a pressure of 400 MPa. In this pressure ranges are calculated the pore size distribution from approximately 3.6 nm until 400 micron (µm).

**^1^H-NMR** spectra were recorded on a 600 MHz spectrophotometer (JEOL JNM-ECZ600R/M1).

**^13^C{^1^H}-NMR** spectra were recorded on a 150 MHz spectrometer (BRUKER AVANCE III 700)

**Estimation of the degree of substitution by NMR:**

Carbons from CCC fragment = I_CH3_ * (5/3) **Eq. 1**

Carbons from backbone = ΣI_CH2_ – I_CH3_ * (2/3) **Eq. 2**

Degree of substitution (DS) = Eq. 1/ Eq. 2 *100 **Eq. 3**

**Elemental analysis** was performed with a Vario EL from Elementar.

**Raman** spectra were recorded using the FT-Raman spectrometer MultiRAM from Brucker with an excitation wavelength of 1064 nm (Nd:YAG laser). The spectrometer is equipped with a germanium detector, which was cooled with liquid nitrogen. All spectra were measured at room temperature, at 50-100 mW laser power and a spectral resolution of 4 cm^–1^. The measuring range was set between 4000 cm^–1^ to 50 cm^–1^.

**Gravimetric measurements** were carried out with an AT460 Delta Range from Mettler Toledo with a readability of 1 mg and a weighing capacity of 405 g.

**UV/VIS spectra** were recorded on a PerkinElmer Lambda 465.

A **peristaltic pump** with a Masterflex L/S computer-compatible digital drive up to 600 rpm and a Masterflex Easy-Load II L/S head pump was used.

# Cryogel Synthesis and Characterization

## Synthesis of PEI-CCC

hPEI (15.0 g, 0.348 mmol) is dissolved in ethanol (350 mL). Chlorocholine chloride (72.6 g, 0.459 mmol) and triethylamine (46.5 g, 0.459 mmol) were added to the solution. The mixture was heated to 60 ºC and kept at this temperature for 24 h. The mixture was then cooled to room temperature, before removing the solvent under reduced pressure. The crude was dissolved in water (500 mL) and purified using tangential flow filtration. The pure polymer was dried by lyophilization obtaining 26.7 g of the final product as a brown solid.

hPEI (^1^H NMR, 700 MHz, D_2_O): δ [ppm] = 2.69 (bs, 1H), 2.64 (bs, 1.61H).

hPEI (^13^C{^1^H}-NMR, 151 MHz, D_2_O): δ [ppm] = 56.2 (0.99C), 53.1 (1.88C), 51.0 (2.88C), 50.8 (0.89C), 47.8 (1.27C), 45.6 (1.73C), 39.9 (1.00C), 37.8 (0.99C). According to Grenda *et al.*^12^ the composition corresponds to 34.0% of 1º amines, 33.2% of 2º amines and 32.8% of 3º amines.

**PEICCC** (^1^H NMR, 700 MHz, D_2_O): δ [ppm] = 3.96 (1.28H), 3.56 (1.34H), 3.22 (10.52H), 2.91 (14.51H), 2.37 (0.26H), 2.09 (0.37H), 1.36 (1.00H).

**PEICCC** (^13^C{^1^H}-NMR, 151 MHz, D_2_O): δ [ppm] = 53.6 (1.46C), 50.3 (11.43C), 49.4 (1.18C), 45.3 (7.74C), 41.9 (0.62C), 38.8 (0.10C), 37.1 (1.78C).

Degree of substitution (DS) using Eq. 1-3: 15%

**Figure S1.** ^1^H-NMR of commercial hPEI in D_2_O.

**Figure S2.** ^1^H-NMR of **PEICCC** in D_2_O.

**Figure S3.** Inversed gate ^13^C{^1^H}-NMR for the quantitative estimation of the different content of amines in hPEI.

**-CH_3_**

**Figure S4.** Inversed gate ^13^C{^1^H}-NMR for **PEICCC**. Trimethylammonium carbons are highlighted and labeled in green, the rest of carbon signals correspond to the -CH_2_- groups from the polymer backbone and CCC fragments.

## Synthesis of Cryogels 1-3 (CG1[Cl]- CG3[Cl])

A solution of **PEICCC** (2 wt%, (m/v)) and glutaraldehyde (2.5 wt% (m/v)) in deionized water were prepared. Both solutions were previously cooled to 0-1 C to prevent crosslinking before freezing. Afterwards, **PEICCC** solution (4.00 g, 200 mL) and glutaraldehyde solution (1.36 g, 54.5 mL) were mixed and stirred vigorously at this temperature until homogenization of the mixture. The resulting solution was poured into aluminum pans (size: 64 mm diameter, 28 mL volume), and introduced into a freezer at a temperature of –18 ºC. The mixture is kept at this temperature for 24 h. Subsequently, the formed discs are thawed to room temperature and remaining solution is decantated. A solution of NaBH_4_ (2 g of NaBH_4_, 2.1 g of NaHCO_3_ and 0.41 g of NaOH in 1 L of H_2_O), used to reduce the formed imines to the corresponding amines, is added to the cryogels. The solution is exchanged 3 times in 2 h. Afterwards, the cryogels are washed with deionized water until pH 7. Finally, the cryogels are frozen and dried using lyophilization.

**Table S1.** Feed compositions and concentration of the solutions used

| **Sample** | **[PEICCC]_I_**  **(wt%)** | **Volume**  **(mL)** | **Mass (g)** | **[GA]**  **(wt%)** | **Volume**  **(mL)** | **Mass (g)** | **[PEICCC]_f_**  **(wt%)** | **mass_PEICCC_/**  **mass_GA_** |
| --- | --- | --- | --- | --- | --- | --- | --- | --- |
| **CG1[Cl]** | 2 | 200 | 4.00 | 2.5 | 54 | 1.36 | 1.57 | 0.34 |
| **CG2[Cl]** | 5 | 80 | 4.00 | 2.5 | 54 | 1.36 | 2.98 | 0.34 |
| **CG3[Cl]** | 10 | 40 | 4.00 | 2.5 | 54 | 1.36 | 4.25 | 0.34 |

## Characterization of Cryogels

### Infrared Spectroscopy

**Figure S5.** IR spectra of hPEI, **PEICCC**, and the cryogels **CG1[Cl]-CG3[Cl]**.

### Thermogravimetric analysis

**Figure S6.** TGA curves for the cryogels **CG1[Cl]-CG3[Cl]**.

### Scanning Electron Microscopy


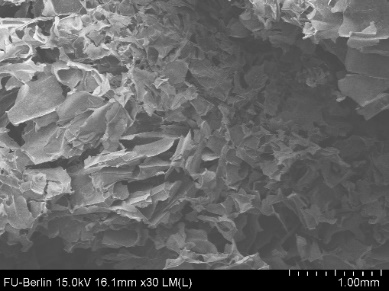

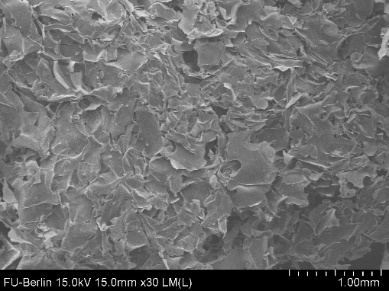

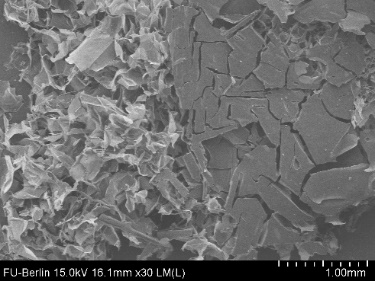


**(CG1[Cl])**

**(CG3[Cl])**

**(CG2[Cl])**

**Figure S7.** SEM images of the cryogels at 30 and 150 magnifications.

**Table S2.** Summary of mercury intrusion porosimetry values.

| **Sample** | **Pore Volume** | **Median Pore Diameter** | **Modal Pore Diameter** | **Porosity** |
| --- | --- | --- | --- | --- |
|  | [mL/g] | [µm] | [µm] | [%] |
| **CG1[Cl]** | 13.672 | 102.1 | 105.1 | 92.3 |
| **CG2[Cl]** | 13.672 | 99.3 | 103.9 | 94.0 |
| **CG3[Cl]** | 9.834 | 93.5 | 97.0 | 91.8 |

# Chlorine Adsorption Experiments

## Conditioning of the Cryogels

Before the chlorine adsorption capacities of the polymers were determined the polymers were dried overnight under reduced pressure at 60 °C. Then, the cryogels were handled under inert conditions by applying argon, performing standard Schlenk techniques and oil pump vacuum up to 10^–3^ mbar. A conditioning of the cryogels **CG1[Cl]**-**CG3[Cl]** and **nq-CG** was performed in which the cryogels were exposed to a chlorine pressure of 1 bar while cooling the sample with a water bath to 20 °C. During this first contact with chlorine a slight weight increase of the polymers was observed as well as a change in color. The initially pale yellow cryogels took a dark yellow color. The chlorine was then removed under reduced pressure at 60 °C over 16 h which again led to a color change of the cryogel into brown.

## Chlorine Adsorption and Desorption Cycles

To run chlorine adsorption, 1 bar chlorine was loaded onto the polymers until the chlorine pressure remained constant which led to the cryogel taking a dark yellow color again. The loading was determined gravimetrically by weighting it and subtracting the weight of the polymer, the mass of the flask under vacuum and the weight of the gaseous chlorine in the volume of the reaction vessel determined by ideal gas law (eq. 4, eq. 5). Again, the chlorine was then removed overnight under dynamic vacuum a 60 °C. This again led to a brown color of the polymer. This cycle was repeated three times without any change in the polymer being detected.

| $m\left( {Cl}_{2\left( stored \right)} \right)=m\left( weighted \right)-m\left( flask \right)-m\left( polymer \right)-m\left( {Cl}_{2 gas-phase} \right)$ | **Eq. 4** |
| --- | --- |
| $m\left( {Cl}_{2 gas-phase} \right)=\frac{p\left( {Cl}_{2} \right)*V_{flask}}{R*T}*M\left( {Cl}_{2} \right)$ | **Eq. 5** |

**Table S3.** Chlorine adsoption capacities of the cryogels **CG1[Cl]-CG3[Cl]** and **nq‑CG**

|  | **Adsorption-Desorption Cycle**  **m(Cl_2_)/m(CG)^[a]^ (g/g)** | | | |
| --- | --- | --- | --- | --- |
| **Cryogel** | **1** | **2** | **3** | **Average** |
| **CG1[Cl]** | 0.29 | 0.24 | 0.22 | 0.25 |
| **CG2[Cl]** | 0.28 | 0.24 | 0.25 | 0.26 |
| **CG3[Cl]** | 0.24 | 0.22 | 0.21 | 0.22 |
| **nq-CG** | 0.16 | 0.16 | 0.15 | 0.15 |

[a] Corresponds to the total amount of Cl_2_ reversibly adsorbed in the cryogel as [Cl_3_]^–^.


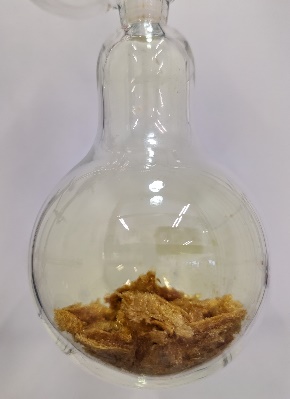

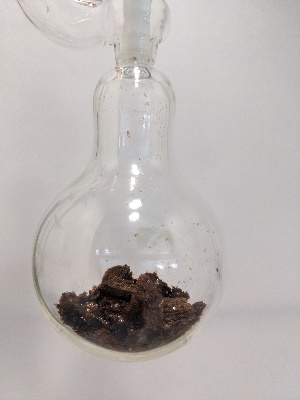

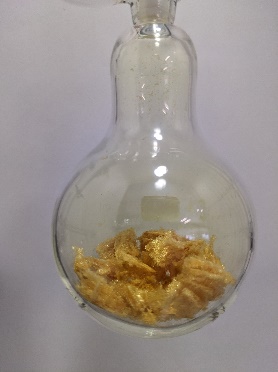


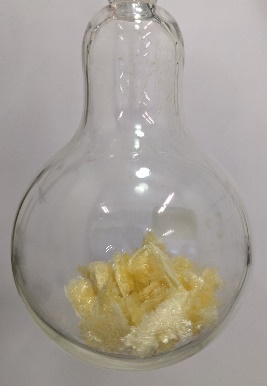


4)

3)

2)

1)

**Figure S8.** Exemplary sequence of the chlorine adsorption desorption cycles. 1) prepared cryogel under air, 2) first exposure to Cl_2_ atmosphere (1 bar), 3) after removing Cl_2_ and 4) second exposure to Cl_2_ atmosphere. Photographs taken by the authors.

### Chlorine Release Process

To investigate the chlorine release process of the cryogel, conditioned and chlorine‑loaded **CG1[Cl_3_]** (3.54 g) was prepared as described above (see 3.2). The amount of stored chlorine was determined gravimetrically (0.648 g). Chlorine was then removed under vacuum (10^–3^ mbar) at 60 °C and the cryogel was weighted after 3 h and 16 h to determine the decrease of weight. The amount of stored chlorine could then be calculated by subtraction of the masses of the loaded and discharged **CG1** with a deduction of the mass of the gaseous chlorine. It was found that 0.407 g (63%) of the chlorine was released after 3 h and 0.466g (72%) after 16 h.

### Elemental Analysis

**Table S4.** Elemental analysis of pristine **CG1[Cl]**, after conditioning and after three chlorine adsorption desorption cycles with its C, H and N proportions in %.

|  | **C** | **H** | **N** | **Others** |
| --- | --- | --- | --- | --- |
| **Pristine CG1[Cl]** | 57.07 | 16.80 | 16.41 | 9.72 |
| **Conditioned** | 33.20 | 7.67 | 9.58 | 49.55 |
| **After** **three chlorine adsorption desorption cycles** | 30.16 | 4.14 | 8.84 | 56.86 |

It must be noted that upon first contact with chlorine the cryogels react partially with chlorine probably due to the chlorination of some non-alkylated amino groups or crosslinker aliphatic chains. Nevertheless, after the initial exposure of the cryogels to chlorine, the reactivity of the crosslinked polymer scaffold towards chlorine is significantly reduced. For instance, the ratio of the carbon to the nitrogen content remains almost constant after both the conditioning and after three chlorine adsorption desorption cycles, which indicates that the chlorination is only happening at the C-H and N-H positions of the crosslinked polymer network.

### Raman Measurements

**Figure S9.** Raman spectrum of pristine **CG1[Cl]** (red) and the chlorine-containing cryogel **CG1[Cl_3_]** (black).

Pristine **CG1[Cl]**: Raman (298 K): 𝜈̃ = 2933 (vs), 2852 (s), 1654 (w), 1452 (m), 1300 (w), 455 (w) cm^–1^.

Chlorine-loaded **CG1[Cl_3_]**: Raman (298 K): 𝜈̃ = 2964 (vs), 1456 (m), 454 (vs, trichloride) cm^–1^.

## Gas Adsorption Experiments with Nitrogen and Oxygen

To perform further gas adsorption experiments with N_2_ or O_2_ the cryogel **CG1[Cl]** was first dried overnight under reduced pressure at 60 °C. Afterwards the cryogel was kept under inert conditions by using standard Schlenk techniques, oil pump vacuum up to 10^–3^ mbar and argon. The cryogel was then exposed to the each gas with a pressure of 1 bar. When the pressure remained constant the amount of adsorbed gas was determined gravimetrically and calculated according to equation 4 and equation 5. The gas phase was then removed overnight under dynamic vacuum and 60 °C and the polymer was weighted again. There was no increase in the weight of the polymer, indicating that oxygen and nitrogen are not adsorbed by **CG1[Cl]**.

## Separation Experiments


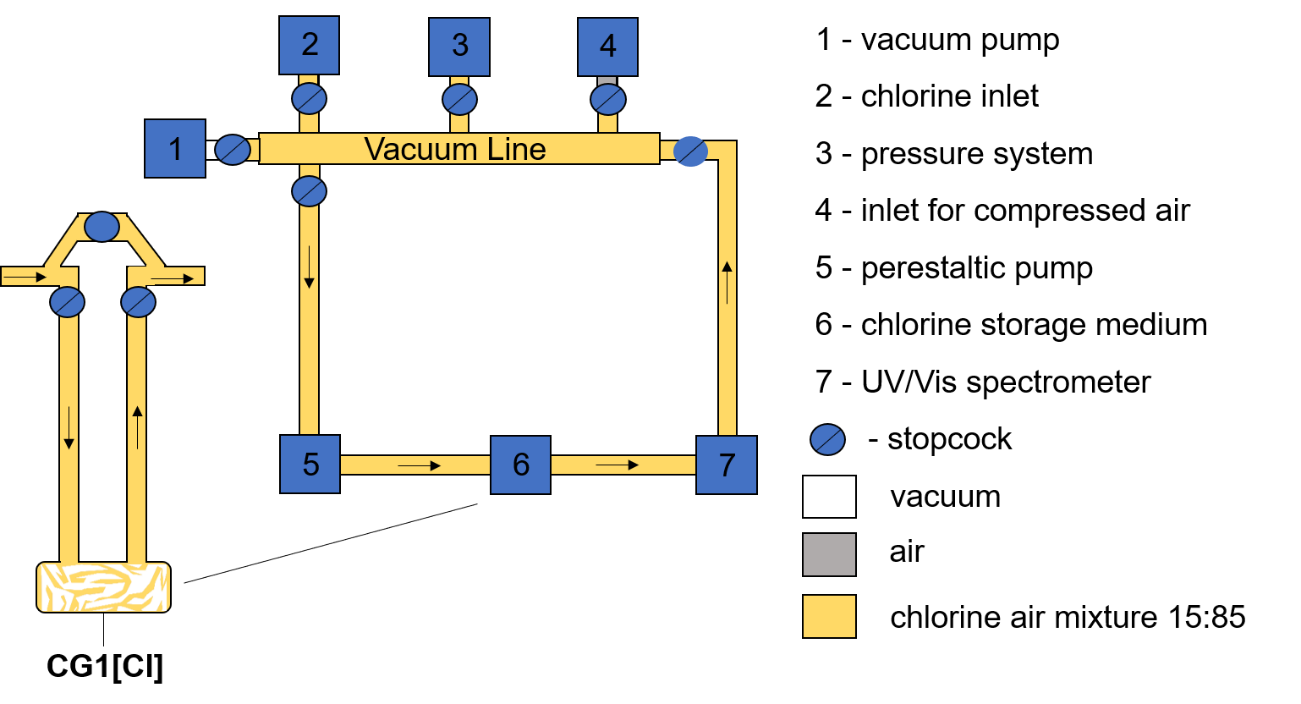


**Figure S10.** Schematic representation of the experimental setup for gas separation experiments.


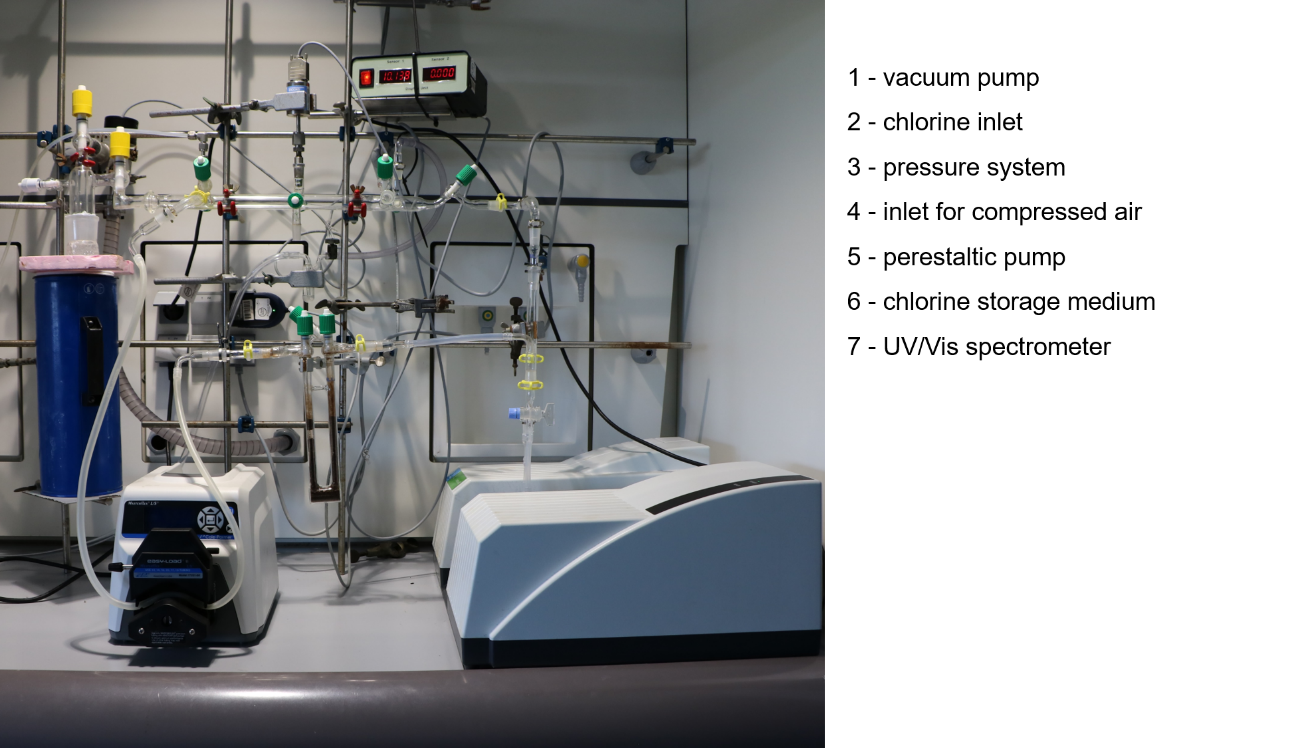


**Figure S11.** Experimental setup for gas separation experiments. Photographs taken by the authors.

To perform gas separation experiments for a mixture of chlorine and compressed air (15:85) a setup (Figure S10 and S11) was constructed consisting of a glass vacuum line, equipped with a chlorine gas and an inlet for compressed air, which is connected to both sides of cooling trap by either perfluoroalkoxy alkane or C-Flex Ultra (Cole-Parmer) tubing to form a circuit. The setup was evacuated and flushed with compressed air twice. Then a gas mixture of chlorine (0.220 g, 150 mbar) and compressed air (850 mbar) was circulated through the empty setup until, after 222 minutes, the gas pressure remained constant. The pressure decrease of 48 mbar indicates a chlorine loss of 32%, while the chlorine loss determined by UV/VIS spectroscopy is 48% for the setup without cryogel **CG1[Cl]** (Figure S13, left).

For the gas separation experiment the cryogel **CG1[Cl]** (1.78 g) was placed inside of the cooling trap, the setup was evacuated and two times flushed with compressed air. Subsequently the premixed gas mixture of chlorine (0.220 g, 150 mbar) and compressed air (850 mbar) was circulated through the system using a peristaltic pump. This was done for 244 minutes until the gas pressure remained constant. The adsorption of chlorine gas was determined by three different methods. First, the amount of adsorbed chlorine in the polymer was determined gravimetrically by weighting the cryogel after the end of the experiment. This method showed a mass increase of 0.096 g which corresponds to a chlorine absorption of 44%. Additionally, the change of pressure within the system was monitored and showed a decrease from 1000 mbar to 886 mbar. Assuming only chlorine is adsorbed, and the pressure of the compressed air remains constant, 114 mbar of chlorine were adsorbed, which corresponds to a chlorine reduction of 76%. Lastly, the kinetics of the chlorine adsorption process was investigated by UV/Vis spectroscopy. The gas mixture was passed through a gas-phase UV/VIS cell and UV/VIS spectra were recorded over the duration of the experiment (Figure S12). The peak area of the chlorine bands was then used to determine the relative absorbance which is decreasing and showed a chlorine absorption of 84% after 244 minutes (Figure S13 left). The absorbance of the UV/VIS measurement of the empty setup was used to correct the absorbance of the gas separation experiment with the cryogel **CG1[Cl]** to provide the corrected chlorine adsorption process over time (Figure S13 right).

**Table S5.** Chlorine adsorption in % of the gas separation experiment determined gravimetrically, by pressure reduction and UV/VIS measurements.

| **Chlorine adsorption (%) determined by** | **Gravimetric measurements** | **Vapor pressure** | **UV/VIS spectroscopy** |
| --- | --- | --- | --- |
| **Blank experiment** | / | 32 | 48 |
| **Setup with CG1[Cl]** | / | 76 | 84 |
| **CG1[Cl] (measured)** | 44 | / | / |
| **CG1[Cl] (calculated)** | 44 | 44 | 36 |

**Figure S12.** UV/VIS spectra of the chlorine absorption in the gas separation experiment with **CG1[Cl]**.







**Figure S13.** Relative absorbance calculated from the peak area of the Cl_2_ band as a function of time for chlorine gas absorption from the empty setup (black) and **CG1[Cl]** (blue) in comparison (left). The absorbance of the gas adsorption experiment with **CG1[Cl]** was then corrected by the adsorption of the empty setup (right).
